# Possibility and Time in Quantum Mechanics

**DOI:** 10.3390/e24020249

**Published:** 2022-02-08

**Authors:** Olimpia Lombardi, Sebastian Fortin, Matías Pasqualini

**Affiliations:** 1 CONICET, Instituto de Filosofía, Universidad de Buenos Aires, Buenos Aires 1420, Argentina; sfortin@conicet.gov.ar; 2 CONICET, Instituto de Investigaciones “Dr. Adolfo Prieto”, Universidad Nacional de Rosario, Rosario 2000, Argentina; matiaspasqualini@gmail.com

**Keywords:** possibility, actuality, parameter-time, event-time

## Abstract

In the discourse of quantum mechanics it is usual to say that non-commuting observables *cannot* have definite values at the same time, or that they *cannot* be simultaneously measured. But, what does the term ‘cannot’ mean in this context? Does it stand for impossible? Should Heisenberg’s principle be read in terms of uncertainty or of indeterminacy? On the other hand, whereas the debates about the nature of time in classical and relativistic mechanics have been many and varied, the question about the nature of time in quantum mechanics has not received the same attention, especially when compared to the large amount of literature on interpretive issues. The purpose of this paper is to show that, under a realist interpretation of quantum mechanics, these two matters, possibility and time, are strongly related. The final aim is to argue that, when possibility and actuality are conceived as irreducible modes of being, they are correlated to two different notions of time that can be distinguished in the quantum realm: parameter-time and event-time.

## 1. Introduction

In the discourse of quantum mechanics it is usual to say that non-commuting observables *cannot* have definite values at the same time, or that they *cannot* be simultaneously measured. But, what does the term ‘cannot’ mean in this context? Does it stand for impossible? Should Heisenberg’s principle be read in terms of uncertainty or of indeterminacy? The answers to these questions depend to a large extent on how the concept of possibility and the complementary concept of actuality are interpreted. Although extremely relevant from a conceptual viewpoint, this issue is not among the most discussed in the field of the foundations of quantum mechanics.

On the other hand, whereas the debates about the nature of time in classical and relativistic mechanics have been many and varied, the question about the nature of time in quantum mechanics has not received the same attention, especially when compared to the large amount of literature on interpretive issues. Perhaps the fact that the symmetry group of quantum mechanics is the same as that of classical mechanics led many to think that any question about time in non-relativistic quantum mechanics is automatically settled by the same discussion in pre-relativistic classical mechanics. However, in the quantum domain the concept of time has some peculiar features that make it different from the corresponding concept in other mechanical theories.

The purpose of this paper is to show that, under a realist interpretation of quantum mechanics, these two matters, possibility and time, are strongly related. The final aim is to argue that, when possibility and actuality are conceived as irreducible modes of being, they are correlated to two different notions of time that can be distinguished in the quantum realm. For this purpose, the article is organized as follows. Section 2 is devoted to the concept of possibility. First, different notions of possibility will be presented. By focusing on possibility *de re*, then the central distinction between possibilism and actualism will be introduced. This distinction will lead us to review the way in which possibility is conceived in different interpretations of quantum mechanics. In Section 3, the concept of time in quantum mechanics will be considered. First, the obstacle to define an observable time will be recalled, and then the difference between parameter-time and event-time will be introduced. In Section 4 we will discuss the link between possibility and time in the quantum domain. In particular, we will argue that the parameter-time is the time of the evolution of possibilities as described by the Schrödinger equation, whereas the event-time corresponds to the domain of actuality, and arises from the relationships between actual events. Finally, in Section 4 we will conclude with some general remarks.

## 2. Modality in Quantum Mechanics

### 2.1. The Different Forms of Possibility

Already in the Middle Ages it was recognized that true propositions are of two types, “necessarily true” and “contingently true”. In other words, there are two “modes” in which a proposition can be true (or false): the mode of contingency and the mode of necessity—hence the term ‘*modality*’. At present, the term is retained to mean “pertaining to possibility and related concepts” (see [1]). In fact, there are other concepts besides “possible” involved in the modal discourse: impossible, necessary, and contingent. However, all of them can be defined in term of *possibility*: *X* is impossible ≡ not (*X* is possible); *X* is necessary ≡ not (non-*X* is possible); *X* is contingent ≡ not (*X* is necessary) ≡ (non-*X* is possible). Therefore, here the focus will be the concept of possibility.

In addition to the above modal concepts, there is another notion that is essential to the understanding of what possibility means: *actuality*. In principle, something that is possible may be realized or not: it may actualize, become actual or not. In other words, the realm of possibility is, in principle, partitioned into possible-and-actual and possible-and-non-actual. The relation between possible and actual is expressed by certain modal intuitions that any conception of possibility should incorporate: (i) whatever is actual is possible, (ii) whatever is necessary is actual.

The very first question is what the predicate ‘possible’ is applied to. At the beginnings of XII century, Peter Abelard seems to be the first in realizing, when studying syllogistics, that possibility may be said in two ways: (i) it may apply to what is expressed by a non-modal sentence (*de dicto*; for example, possibly ‘every A is B’), or (ii) it may express the mode through which the predicate belongs to the subject and, therefore, is associated with the thing (*de re*; for example, ‘every A is possibly B’) (see [2,3]). In contemporary terms (see, e.g., [4]) it is said that *modality de dicto* applies to linguistic items, in particular, to propositions and, thus, it is studied by logics, whereas *modality de re* applies to ontic items (objects, properties, states of affairs), so it *belongs to ontology.*

Related to but different from the distinction *de dicto-de re*, another classification expresses the different forms of modality: semantic, subjective and objective modality. *Semantic modality* is relative to a logical system *S* and is defined in terms of derivability in the context of *S*: a proposition *p* is semantically possible relative to a logical system *S* iff ¬ *p* is not derivable from *S*, and it is semantically necessary relative to *S* iff it is derivable from *S* [4]. *Subjective or epistemic modality* is relative to an agent and the evidence possessed by that agent, and it is defined in terms of evidence and ideal reasoning: a proposition *p* is subjectively necessary for an agent *A* iff the evidence *E_A_* that *A* possesses and ideal reasoning are sufficient to rule out ¬ *p* [1]. *Objective modality* is an ontological category, which refers to how reality may be and, then, is closely related with modality *de re*: modal concepts apply to ontic items (objects, properties, states of affairs) independently of knowledge and agents, because objective modality is a mode of being of reality in itself. In general, *de dicto* use of modality is linked with semantic and subjective modality. Nevertheless, it may be the case that the *de dicto* use of modality expresses *de re* modality: a proposition *p* is *de dicto* possible because the fact semantically referred to by *p* is objectively-*de re* possible.

Despite the short life of logical positivism, its great influence extended throughout the entire 20th century. Its rejection of metaphysics in favor of logic led most philosophers of science belonging to the analytic tradition to avoid ontological discussions by translating them into linguistic and logical questions. It is not surprising, then, that this general position showed a deep skepticism towards any appeal to objective modality, manifested by the *de re* use of modal concepts. Perhaps the most conspicuous enemy of objective modality was Willard V. O. Quine [5]. According to him, if modality *de dicto* made any sense, it should be understood in terms of the concept of analyticity, in terms of what we called ‘semantic modality’. But since modality *de re* cannot be understood in terms of analyticity it cannot be understood at all.

Many authors reject objective possibilities on the grounds that there is no non-trivial identity criterion for non-actual items; this is again the case of Quine [6]. In order to face this challenge, different strategies aimed at the identification of possibilities have been developed: possible worlds, subsistence as different from existence, etc. These discussions consider examples such as the possibility of the existence of Vulcan as a planet closer to the Sun than Mercury, or the possibility that Julius Caesar had a sixth finger on his right hand. But these are not situations that are relevant to the treatment of the problem of possibility in physics: no all-encompassing theory of possibility is required in the physical domain, which could be applied to any kind of possible fact—under the assumption that this is possible. The notion of objective possibility relevant here is the one that refers to the facts described by physics: it is physical theories that define the space of objective possibility. In what follows we will confine the discussion to this type of possibility, which we will call *physical possibility*: a *de re* objective possibility, embodied in the laws of physics.

An example of the rejection of metaphysics in favor of logic is the case of Richard Montague [7], who attempts to elucidate the concept of physical necessity in logical terms. According to this author, *p* is physically necessary iff *p* is deducible from a certain class of physical laws *L*. This view conflates necessity with lawfulness. However, regardless of the way in which natural laws are philosophically conceived, this is not the way in which laws are understood with respect to possibility in the practice of physics: laws do not fix what is to necessarily happen, but only what may happen. What actually happens depends not exclusively on the laws but also on certain particular circumstances of the phenomenon in question that do not depend on the laws. The paradigmatic case is that of dynamic laws, which only establish the evolutions that a system may describe; but which of all the possible evolutions will be actualized depends on the initial conditions and the boundary conditions of the particular situation. In other words, a physical theory distinguishes the realm of possibility from the realm of impossibility. For example, special relativity makes it impossible for a body to have a velocity higher than that of light. Within the realm of possibility, physical theories admit some facts as necessary, for example, those referring to certain elementary properties such as the electric charge of the electron or the mass of certain subatomic particles. But this does not imply that lawfulness must be identified with necessity.

It is just since the last decades of the twentieth century that the philosophy of science begun to get free from the strong ties imposed by logical positivism and its descendants. In particular, philosophers of physics began to consider the problems related with the ontic reference of physical theories. When this task focuses on modality, the need of recovering an objective meaning of modality comes to the fore.

### 2.2. Actualism Versus Possibilism

The problem of the nature of objective possibility is as old as philosophy itself. It already appears in Aristotle’s discussion of future contingents. The question was how to assign a truth value today to statements about the future, such as ‘There will be a sea-battle tomorrow’ and ‘There will not be a sea-battle tomorrow’: according to Aristotle, while the past is fixed, the future is open to possible alternatives. In the generation after Aristotle, the Megarian logician Diodorus Cronus devised an argument to show that there can be no future contingency at all: the future is as fixed as the past, there are no real possibilities (see [8]). This ancient discussion brings out two different conceptions of modality.

*Actualism* is the view according to which everything that exists, everything that in any sense can be said to be, is actual. In other words, actualism denies that there is any kind of being beyond actual existence: to be is to exist, and to exist is to be actual (see [9]). This was precisely the position of Diodorus Cronus; in Cicero’s words, “Diodorus defines the possible as that which either is or will be” (quoted in [10]: 117). According to Diodorus, what actually happens is all that *can* happen. Therefore, any claim about possibility must be reduced to a claim about actuality. For Arthur Prior, Diodorus “seems to have been an ancient Greek W.V. Quine, who regarded the Aristotelian logic of possibility and necessity with some skepticism, but offered nevertheless some «harmless» senses that might be attached to modal words” ([11]: 16). This view survived through the centuries until contemporary times; for instance, for Bertrand Russell ‘possible’ means ‘sometimes’, ‘necessary’ means ‘always’, and ‘impossible’ means ‘never’ ([12]: 165).

*Possibilism* is the denial of actualism. It is the thesis that there are ontic items (things, properties, states of affairs) that are not actual. As a consequence, possibility is an irreducible ontological category. This conception is exemplified in Philo of Megara, for whom possibility is an intrinsic capability of the propositions to be or not to be true or false. In Boethius words, for Philo “possible is that which is capable of being true by the proposition’s own nature” (quoted in [13]). In his dispute with Diodorus, the Stoic philosopher Chrysippus also adopts a possibilist position when he maintains that “even though something is not true and may never be true, it may nevertheless be possible” ([14]: 77–78); so, possible is defined as “that which is not prevented by anything from happening even if it does not happen.” (quoted in [4]: 172).

The debate actualism versus possibilism is still alive in present-day metaphysics, but in general it is centered on the possible existence of individuals: according to actualists, all possible individuals are actual, whereas possibilists admit the existence of *possibilia*, that is, non-actual possible individuals. However, when the interest is focused on modality in physics, particular individuals play no role: physics is not interested in this or that, say, electron, but in electrons in general. Therefore, it is convenient to let aside the discussions of contemporary metaphysicians and come back to Antiquity. For actualists, the concepts of actuality and possibility are coextensive: possibilities that never happen are impossible; thus, possibility must be reduced to actuality. For possibilists, on the contrary, actuality is a subclass of possibility: there are possibilities that may never become actual. This means that, for them, possibility is an irreducible ontological category: reality unfolds in two domains, the domain of possibility and the domain of actuality.

When these two conceptions of the relation between possibility and actuality are clearly distinguished, it is not difficult to perceive again the influence of logical positivism on twentieth-century philosophical thought. According to the verificationist theory of meaning, a sentence is meaningful only when its truth or falsity can, at least in principle, be determined by observation. It is quite clear that if a modal sentence refers to merely possible states of affairs, its truth value cannot be determined by observational means: this explains the rejection of possibilist possibility in traditional philosophy of science.

This actualist view of possibility can be easily observed in the twentieth century treatment of determinism, the basic idea of which can be found in William James words: “The future has no ambiguous possibilities hidden in his womb” ([15]: 150). However, as soon as actualist philosophers attempt to pin down the concept, they fall into positions that oscillate between emptiness and triviality. On the basis of the verificationist theory of meaning, the notion of determinism is meaningless if it is not defined in terms of an empirical criterion that unequivocally establishes whether a system is determinist or indeterminist. For this reason, the notion of determinism has usually been identified with that of predictability. For instance, Karl Popper proposes a “scientific” concept of determinism as “the doctrine that the state of any closed physical system at any given future instant of time can be predicted, even from within the system, with any specified degree of precision” ([16]: 36). This claim, which conflates epistemology with ontology, renders the notion of determinism completely empty, because virtually the state of any physical system becomes unpredictable after a sufficiently long time and under sufficiently unstable conditions. On the other hand, in an attempt to purify the notion of determinism of epistemic components, Bertrand Russell states that “[a] system is said ‘deterministic’ when, given certain data *e*_1_, *e*_2_, …, *e_n_* at times *t*_1_, *t*_2_, …, *t_n_* respectively, concerning this system, if *E_t_* is the state of the system at any time *t*, there is a functional relation of the form *E_t_* = *f*(*e*_1_, *t*_1_, *e*_2_, *t*_2_, …, *e_n_*, *t_n_*).” ([17]: 398). However, as the author himself acknowledges, this definition trivializes the notion of determinism, since it is always possible to define a function that “passes” through a finite number of points. To avoid this conclusion, Russell needs to add additional requirements, such as the simplicity of *f* or the condition that *f* does not explicitly depend on *t*.

Perhaps the most recent manifestation of actualism in the philosophy of science is Humeanism regarding laws of nature, according to which laws are just patterns in the mosaic of actual events. This view is inspired in David Lewis’s “Humean supervienience”, that is, “the doctrine that all there is to the world is a vast mosaic of local matters of particular fact, just one little thing and then another.” ([18]: ix). According to Lewis [19], among all the deductive systems that make true claims about the “Humean mosaic”, the best system is the one that reaches the best balance between simplicity, strength, and fit. A natural law is a theorem in the best system (for criticisms, see, e.g., [20]). It is quite clear that, in this picture, there is no room for non actualized possibilities.

Despite the predominance of the actualist position in the current philosophy of science, the question is whether this point of view agrees with the way in which practitioners of physics conceive physical laws. The answer is that a concept of objective possibility irreducible to actuality seems to be pervasively present in the discourse of physics. When a physicist asserts the impossibility of superluminary signals, she is not merely asserting their de facto non-existence: such signals cannot exist in a reality described by relativistic laws. In turn, it cannot be guaranteed that everything that is physically possible will become actual at some point in time: for a possible event to actually occur, certain circumstances must be present, without which the event remains within the realm of possibility forever. For example, according to statistical mechanics, it is possible for all the molecules of a gas confined in a container to spontaneously agglomerate at one of its corners, although it may be the case that this never occurs in the entire history of the universe. The transition from the possible to the actual from the perspective of physics has much more in common with the Aristotelian categories of potency and act than it shares with the traditional philosophy of science and its actualist interpretation of possibility.

### 2.3. Possibility, Probability, and Interpretation of Quantum Mechanics

After more than a century from Planck’s first description of a non-classical phenomenon, quantum mechanics remains a severe challenge for philosophers of physics and physicists interested in foundational issues. One of the peculiarities of the theory is that the quantum state does not specify definite values of the observables of a system, but rather assigns probabilities to those definite values. Perhaps for this reason, the interest has been mainly focused on understanding probability, while the concept of possibility has been much less addressed in the field of the interpretation of quantum mechanics. However, if probability is conceived in some sense as a measure of possibility, it is easy to see that there is a correspondence between the different interpretations of the concept of probability and those of the concept of possibility.

According to a well-documented study [21], our modern conception of probability has had dual nature since its emergence in the mid-seventeenth century: in its *epistemic* sense, it measures the degree to which evidence supports a given hypothesis; in its *ontological* sense, it describes indeterministic regularities exhibited in nature. In the twentieth century, two widely recognized analyses of epistemic probability have been proposed (see [22]). According to the *logical* interpretation —the tradition of John Keynes [23] and Rudolf Carnap [24]—probabilities can be determined by the examination of the space of possibilities, and express the degree of support that a piece of evidence confers to a given hypothesis. According to the *subjective* interpretation —represented by Frank Ramsey [25] and Bruno de Finetti [26]—probabilities measure degrees of confidence or belief of suitable agents. In turn, ontological probability appeared originally under two different forms. The *frequentist* interpretation—e.g., Hans Reichenbach [27] and Ludwig von Mises [28]—identifies probabilities with relative frequencies in infinite sequences. In turn, since the 1950s, the idea of interpreting ontological probabilities as *propensities*—Karl Popper [29]—began to be considered by some of philosophers as an alternative to the frequentist interpretation. Recently, the Humeanist so-called *best-system* interpretation shows some similarities with the frequentist approach, but tries to avoid its main difficulties: inspired by Lewis’s conception of laws of nature [19], probabilities are defined by those probabilistic laws that turn out to be the best account of the Humean mosaic according to simplicity, strength, and fit.

When these interpretations of probability are compared to the different views on possibility as introduced in the previous subsection, it is not difficult to see a conceptual match. On the one hand, *epistemic* probabilities can be conceived as the counterpart of de dicto possibilities. In this epistemic context, the *logical* interpretation corresponds to *semantic* possibility, while the *subjective* interpretation corresponds to *subjective* possibility. On the other hand, *ontological* probabilities are the counterpart of *de re* and *objective* possibility. In this context, the *frequentist* and the *best-system* interpretations implicitly presupposes an actualist view of possibility, whereas the *propensivist* interpretation relies on a *possibilist* conception of possibility.

The strong influence of the positivist’s rejection of metaphysics, expressed by an excessively strict conception of meaning, led to a non-possibilist conception not only of possibility but also of probability. This conception would be natural if physical theories were still formulated in a deterministic framework with reducible probabilities. But this is not the case since the advent of quantum physics. However, many approaches to quantum mechanics are still based on that non-possibilist view.

Although the Copenhagen Interpretation, is not a single, definite doctrine (for a critical historical overview, see [30]), its different versions share certain points about how possibility and probability enter the scene. In particular, quantum mechanics is considered an indeterministic theory, in the context of which the wave function expresses a probability amplitude. However, probabilities have a positivistic flavor. On the one hand, they are always referred to measurements: probabilities are not simply manifested by the frequencies of the measurements’ outcomes, but are defined in terms of those frequencies. On the other hand, as a result of an operationalist influence, all variables are ill-defined unless they refer to measurement outcomes; therefore, asking for probabilities independently of the experimental set-up is meaningless because it is deprived of operational meaning.

In contrast to the Copenhagen view, Bohmian Mechanics is a deterministic theory. In its framework, the complete description of a quantum system requires not only the wave function, which evolves according to the Schrödinger equation, but also the specification of the actual positions of the particles, which evolve according to a guiding equation that expresses the velocities of the particles in terms of the wave function (for updated presentations, see [31,32]). In this context, the ultimate justification for probability distributions is given in terms of statistical patterns exhibited by ensembles of the actual subsystems of the universe, which are probabilistically distributed according to the Born rule. In other words, Bohmian mechanics endows probabilities with an epistemic, “ignorance” meaning, as in the case of the probabilities involved in classical statistical mechanics. 

At present there are many versions of the Many Worlds Interpretation; nevertheless, some features are common to all of them (see [33] for an updated presentation). The state vector never collapses: with each measurement (or interaction), the entire world splits into many copies, each of which “contains” one of the components of the superposition, and from then on each copy follows its own evolution. Although not sufficiently emphasized, under this interpretation quantum mechanics is a deterministic theory, according to which all alternatives are actual (see [34]). Consequently, possibility and probability must be interpreted as epistemic, in terms of the ignorance of an observer confined to only one of the multiple worlds.

Quantum Bayesianism or Qbism, led nowadays by Christopher Fuchs, originated as a point of view on probabilities in quantum mechanics ([35]; see [36] for an updated presentation). QBists claim that the quantum state is not the representation of a physical system, but rather expresses the subjective state of belief of an agent regarding possible future experiences. Therefore, they explicitly adopt a subjectivist interpretation of probability inspired by the works of de Finetti. This perspective makes the Born rule not a law of nature but an empirically motivated rule that a rational agent must follow in order to win when betting on quantum systems.

According to Wave Function Realism, the wave function is a concrete object, a physical field on configuration space. However, in this case the configuration space is not an abstract space used to represent possible configurations of particles in the three-dimensional space, but rather it is the physical space of the universe. In this multidimensional space, material objects are classical configurations of particles that correspond to regions in which the amplitude of the wave function is high ([37]; see also [38]). This means that our impression of living in a three-dimensional space is somehow flatly illusory. Furthermore, since the wave function evolves deterministically in the fundamental multidimensional space, probabilities also turn out to be subjective “illusions”.

This very quick survey of some of the currently discussed interpretations of quantum mechanics shows how difficult it still seems to conceive of a possibilist possibility and a correlative propensity probability in this philosophical field. Some of these interpretive views design different strategies to make probability a measure of the observer’s ignorance about an underlying reality that is inherently deterministic or, in the case of qbism, a mere playing field for betting. Only the most traditional Copenhagen interpretation endows probability with an objective content, but with an actualist-frequentist conception in terms of measurement outcomes. The challenge seems to be, then, to devise an interpretation of quantum mechanics based on a possibilist conception of possibility. In Section 4, two interpretations that explicitly take this stance will be introduced.

## 3. Two Notions of Time in Quantum Mechanics

### 3.1. The Obstacle to an Observable Time

In his original paper on uncertainty relations, Werner Heisenberg [39] was concerned with the way in which the minimization of the error in a position measurement involves a larger disturbance in the momentum measurement. On the basis of the Fourier analysis of wave packets, “uncertainty” relations between position and wave number (spatial frequency), and between time and (time) frequency, can be formulated: ΔxΔk≥1/2, ΔtΔω≥1/2. By appealing to the de Broglie relations, p=ħk and E=ħω, shortly after the publication of Heisenberg’s paper, Niels Bohr [40] proposed uncertainty relations for position-momentum and time-energy with the same status:

(1) ΔxΔp≥ħ/2  ΔtΔE≥ħ/2.

Since then, debates about the meaning of the time-energy uncertainty relation became ubiquitous in the quantum foundations literature, with no general agreement (for a thorough discussion, see [41,42]; see also [43]).

The main obstacle to conceiving the uncertainty relation for position-momentum and the uncertainty relation for time-energy on an equal footing is that, while position, momentum, and energy are represented by operators, time is represented by a scalar; in other words, time is not an observable of the quantum system. This fact caused perplexity since the early days of quantum mechanics. For instance, John von Neumann considered it “the chief weakness of quantum mechanics: its nonrelativistic character, which distinguishes the time *t* from the three space coordinates *x*, *y*, *z*, and presupposes an objective simultaneity concept. In fact, while all other quantities (especially those *x*, *y*, *z* closely connected with *t* by the Lorentz transformation) are represented by operators, there corresponds to the time an ordinary number-parameter *t*, just as in classical mechanics.” ([44]: 354).

The natural strategy to overcome this difficulty would be to define a time observable T that would allow obtaining the uncertainty relation for time-energy as a particular case of the general relation between any two observables A and B—whose domain of definition has to be specified in each case—known as “Heisenberg-Robertson uncertainty principle” (see, e.g., [45]: 223–224):

(2) $$\Delta_{\rho }A\cdot \Delta_{\rho }B\geq \frac{1}{2}\left|{\langle \left[A,B\right]\rangle }_{\rho }\right|=\frac{1}{2}\left|{\langle AB-BA\rangle }_{\rho }\right|,$$

where ρ is the system’s state and $\Delta_{\rho }O^{2}={\langle O^{2}\rangle }_{\rho }-{\langle O\rangle }_{\rho }^{2}$. Regarding the possibility of defining a time operator, it is worth recalling a well-known historical episode. In one of his famous articles from the 1920s, Heisenberg [39] defined a time operator T, conjugate to the Hamiltonian, and formulated the corresponding uncertainty relation. However, some years later, Wolfgang Pauli [46] proved a theorem according to which the fact that any Hamiltonian is bounded from below precludes the existence of a self-adjoint operator T acting as a generator of a unitary group representation of translations in the energy spectrum.

Given the limitation imposed by Pauli’s theorem, the time-energy uncertainty relation was formulated by appealing to different resources. For example, let us consider a generic observable R, incompatible with energy, which acts as an observable correlate of time ([47]; for a simple presentation, see [45]: 344–345). The characteristic time $\tau_{R}$ is defined as

(3) $$\tau_{R}=\frac{\Delta_{\rho }R}{\left|\frac{d}{dt}{\langle R\rangle }_{\rho }\right|}.$$

By combining the Heisenberg equation iħdR/dt=[R,H] with the general uncertainty relation of Equation (2), the definition of $\tau_{R}$ leads to

(4) ΔτR ΔρH≥ħ2.

However, the characteristic time $\tau_{R}$ cannot be considered a perfect substitute for time t, since it depends on the particular observable R (for a different approach, see [48,49]).

### 3.2. Parameter-Time and Event-Time

In classical mechanics, the state of a system is defined in terms of the actual properties that the system acquires over time. In quantum mechanics, by contrast, the state only provides probabilities on the possible values of the properties of the system, while such properties acquire current values only at some specific points in time (this view excludes Wave Function Realism according to which the wave function is a concrete physical field in a high dimensional space). Therefore, at least two different notions of time need to be distinguished in the quantum realm. In Carlo Rovelli’s words: “There are two independent notions of time in ordinary quantum mechanics: the time in which the system evolves, and the «time» that orders the measurements of the observer. These two are not related and may be non-coincident.” ([50]: 130). More precisely:

- The *parameter-time* is the time over which the system’s state unitarily evolves. It is represented by the variable *t* as it appears in the Schrödinger equation.
- The *event-time* (also called ‘observable’ time by Paul Busch [42]) is the time at which particular events occur. Those events are measurement results or, more generally, any acquisition of a definite value by a certain observable.

The parameter-time is the notion of time involved in the characterization of the Galilean group; thus, in quantum mechanics it is supposed to be homogeneous and isotropic as in the classical case. The Schrödinger equation rules how the probabilities on the possible values of all the observables of the system change along the parameter-time. In his detailed works on the meaning of the uncertainty principles, Busch [41,42,51] introduced a further distinction on the basis of how the parameter-time is measured. The *external time* is that measured by a clock that is not dynamically connected with the system studied in the experiment. This time acts as an external parameter of the system’s evolution. The *intrinsic time*, by contrast, is defined through the dynamical behavior of the quantum systems themselves: every *dynamical variable* of a physical system can be used to mark the passage of time. In other words, in principle every non-stationary quantity A defines, for any initial quantum state ρ, a characteristic time $\tau_{\rho }(A)$ within which the expectation value A changes significantly.

Although both external and intrinsic times make sense in the context of local non-relativistic quantum mechanics, when the focus is quantum gravity, the idea of an external time turns out to be at least controversial. Moreover, as many authors emphasize (e.g., [52,53]), one of the main obstacles to the formulation of a quantum theory of gravity is the difference between the notions of time in quantum theory and in general relativity, a theory invariant under general coordinate transformations. For these reasons, some authors take the perspective of the intrinsic time and attempt to reconstruct it from correlations. For instance, Don Page and William Wootters ([54], see also [55]) propose an “evolution without evolution”. Their central idea is that the universe is a stationary scenario and that time and evolution emerge in a subsystem of the universe, which is entangled with another subsystem that satisfies the conditions to be considered a clock. In turn, Rovelli [56,57,58] generalizes the Heisenberg picture by distinguishing between traditional observables and partial observables, which correspond to precise measuring procedures. Since, for Rovelli, all partial observables are on the same footing, dynamics expresses the relation between partial observables, one of which may be selected as a clock (We are grateful to an anonymous reviewer for pointing out a different approach to, in Isham’s terms, “the problem of time in quantum gravity”, in particular, the embedding of the Stueckelberg-Horwitz-Piron program [59] into the framework of general relativity [60]. In this framework, the conflict between external time and intrinsic time in general relativity might be faced by conceiving the parameter time as an invariant universal “world time” ([60]: 2)).

In contrast to the parameter-time, the event-time has no formal representation in quantum mechanics. Yet it is essential in order to endow the theory with physical meaning: testing the theory is only possible by recording specific events, such as the hit of an electron on a screen or the absorption of a photon by an atom, which occur at particular times. The absence of a formal representation perhaps explains why, despite its central relevance, the nature of the event-time and its relationship with the parameter-time has been scarcely discussed in the literature on quantum foundations (as exceptions, see [61,62], where a relational reconstruction of the event-time is proposed). In what follows, the difference between parameter-time and event-time will be related to the modal dimension of quantum mechanics.

## 4. The Link between Possibility and Time

### 4.1. Possibility and Parameter-Time

Although most approaches to quantum mechanics adopt either a subjective or an objective but actualist reading of possibility and probability, two interpretations show that there is room for a possibilist view that endows modality with an ontological content.

According to the Transactional Interpretation of quantum mechanics (TI), originally proposed by John Cramer [63,64], quantum interactions are transactions. An “emitter” generates a retarded wave function forward in time (an offer wave OW, represented by the usual quantum state |Ψ〉), and also an advanced wave function backward in time; an “absorber” responds by generating a retarded wave function forward in time and an advanced wave function backward in time (a confirmation wave CW, represented by the dual state 〈Φ|). A transaction event occurs when the confirmation wave CW from the absorber exactly reinforces the offer wave OW from the emitter, and exactly cancels both the advanced field from the emitter and the retarded field from the absorber; thus, all that remains is a fully retarded wave carrying energy from the emitter to the absorber. This proposal was endowed with a clear possibilist content by Ruth Kastner; the *Possibilist Transactional Interpretation* (PTI) explicitly announces “the reality of possibility” from the very title of Kastner’s 2013 book. According to this view, when an emitter generates an OW, many different transactions are possible, among which only one of them actualizes. But the central point is that possibility is conceived as physically real: “OW and CW are interpreted ontologically in PTI as *physically real possibilities*. In this context, «real» means physically efficacious but not necessarily *actualized*” ([65]: 68; italics in the original). In other words, “the dynamical possibilities referred to by state vectors in PTI are Heisenbergian potentia” ([65]: 68; see also [66]). In turn, the actualization of one of the possible transactions is an irreducible stochastic phenomenon, which “is not compatible with any causal process within the confines of ordinary spacetime.” ([65]: 68).

The Modal-Hamiltonian Interpretation (MHI) is a member of the modal family of interpretations of quantum mechanics. It is a realist and no-collapse approach that endows the Hamiltonian *H* of a closed system with an essential role: the preferred context, that is, the definite-valued observables of the system, is composed by *H* and all the observables commuting with *H* and having, at least, the same symmetries as *H* ([67]; for a Galilean invariant version, see [68]). The MHI supplies an account of the measurement problem, both in its ideal and its non-ideal versions, and has been applied to several well-known physical situations (free particle with spin, harmonic oscillator, free hydrogen atom, Zeeman effect, fine structure, the Born-Oppenheimer approximation), leading to results consistent with empirical evidence. More recently it has extended its applications to further situations, such as the non-collapse account of consecutive measurements in physics [69] and the problem of optical isomerism in chemistry [70]. The point to emphasize here is that, due to its modal nature, for the MHI the formalism of quantum mechanics does not determine what actually is the case, but rather describes possible events with their corresponding probabilities. But the specificity of the MHI is its ontological turn in the conception of modality, since possibility is endowed with a possibilist, non-actualist interpretation: “for each definite-valued observable, among all the possibilities described by the theory, only one is actually realized: the remaining possibilities do not become actual, and they might never become actual in the particular system under consideration.” ([67]: 426).

Despite their differences, the PTI and the MHI agree in that the wave function (or, better, the vector state in any of its representations) represents objective probabilities, which measure the propensity or potentiality to actualization of the possible events. Although those possibilities and the corresponding probabilities are not reducible to actuality, they are real. In other words, reality unfolds in two realms: possibility and actuality. The Aristotelian *dictum* about being can be applied to this case: being can be said in different ways, as possible being or as actual being, neither of which can be defined in terms of the other. When one conjures up the prejudice that reality is exhausted by actual reality, one can realize that, strictly speaking, both the PTI and the MHI advocate a “wave function realism” or, better, a “vector state realism”: the quantum state describes reality in its possibility domain.

The fact that potentialities belong to the realm of possibility does not mean that they do have no physical consequences in the realm of actuality. On the contrary, they produce definite effects on actual reality even if they never become actual. An interesting manifestation of such effectiveness is the case of the so-called “interaction-free measurements”, in which the presence of a quantum system can be detected without any interaction between it and the measuring device. A particular case of such experiments is the Elitzur-Vaidman bomb tester [71]: by finding its roots in the double-slit experiment, in this case non-actualized possibilities are used to test bombs without exploding them. 

From this possibilist perspective, then, the quantum state does not refer to actual events, but describes possibilities, measured by objective probabilities: it “lives” in the realm of possibility. Therefore, its time-evolution also occurs in the realm of possibility: the Schrödinger equation encodes how those probabilities change over time. But, as explained in the previous section, the time of the unitary evolution of the state is the parameter-time. This means that the parameter-time is the time over which possibilities evolve and, consequently, it is what endows the possibility realm with temporality. Nevertheless, we have access to potentialities only through actual events. As a consequence, the realm of actuality enters the scene.

### 4.2. Actuality and Event-Time

Any interpretation that postulates the actualization of certain events as a phenomenon that is not merely epistemic, but objective, is committed to specifying under what circumstances actualization occurs. In some versions of the Copenhagen interpretation, the collapse of the wave function is conceived as a sort of actualization linked to the act of measurement: collapse happens when the quantum system interacts with a macroscopic device or when a conscious being becomes aware of the result of the measurement. In the GRW version of quantum mechanics, collapse is a physical indeterministic phenomenon that occurs repeatedly and spontaneously with a probability 1/*t* per second, where *t* is a new constant of nature. Here we will focus on the two possibilist interpretations introduced above: the PTI and the MHI.

Let us consider a general situation according to the PTI: an emitter atom S generates an OW labeled |s〉, which encounters absorber atoms labeled *A*, *B*, etc., giving rise to different possible or “incipient” transactions (see [65]: 53). In this situation, the OW |s〉 decomposes into different components, each one also an OW. The component of |s〉 absorbed by *A* is 〈a|s〉|a〉, the component of |s〉 absorbed by *B* is 〈b|s〉|b〉, and so on. In turn, the absorbers *A*, *B*, etc. respond with the advanced CWs 〈s|a〉〈a|, 〈s|b〉〈b|, etc., respectively. The product of the OWs’ and the CWs’ amplitudes gives the Born Rule for the probability of the corresponding outcome, e.g., $p(A/S)=\langle s|a\rangle \langle a|s\rangle ={\left|\langle a|s\rangle \right|}^{2}$. Among the collection of those *N* possible transactions, only one becomes actual as a consequence of an irreducibly indeterministic process, say, the transaction linking atoms *S* and *A*, which, as a result of this actualization, are ready to participate in new possible transactions. For instance, atom *A* can become a new emitter, which generates an OW labeled |a〉, which encounters absorber atoms labeled *B’*, *C’*, *D*’, etc., and the process repeats. Therefore, the actual transaction-events resulting from the diverse encounters between emitters and absorbers are related to each other so that they form a network. It is precisely this network of actual events what constitute space-time: “In this interpretation, spacetime is no more—and no less—than the set of actualized transactions” ([65]: 73). In other words, the PTI assumes a relationalist conception of space-time such that “the term ‘spacetime’ describes the structured set of events themselves” ([65]: 75; for a detailed account of the emergence of space-time in the PTI, see [62,72]).

In the case of the MHI, a closed quantum system is a bundle of type-properties—represented by the system’s observables—each one with its possible case-properties—represented by the eigenvalues of each observable. Among the type-properties of the bundle, only those of the preferred context—the definite-valued observables defined by the Hamiltonian—acquire an actual case-property—the actual definite value of each definite-valued observable. Among all the case-properties of each type-property of the preferred context, only one becomes actual in an irreducibly indeterministic way (see [73]). Since the definite-valued observables of the preferred context always commute with the Hamiltonian, they are constants of motion of the closed system. Thus, actualization events —where each event is the acquisition of a definite value by a definite-valued observable— occur only once in the quantum system, at the constitution of the closed system as such. From the viewpoint of the parameter-time, since then there is no change in the realm of actuality: the definite-valued observables with their corresponding definite values remain unchanged during the entire “parameter-lifetime” of the system. However, in the actuality realm, time itself is constituted by the relations between events. For instance, two systems, *A* and *B*, with Hamiltonians $H^{A}$ and $H^{B}$, respectively, interact to constitute the system *C*, with Hamiltonian $H^{C}=H^{A}⊗I^{B}+I^{B}⊗H^{A}+H_{\mathrm{int}}^{AB}$. Such an interaction leads to the actual event by which the Hamiltonian $H^{C}$ acquires an actual definite value, say, $\omega_{k}^{C}$—and analogously the remaining observables of the preferred context. System *C*, in turn, interacts with a system *D*, with Hamiltonian $H^{D}$, to constitute the system *E*, with Hamiltonian $H^{E}=H^{C}⊗I^{D}+I^{D}⊗H^{C}+H_{\mathrm{int}}^{CD}$, which acquires an actual definite value, say, $\omega_{k}^{E}$. But it also may happen that this second interaction ceases, so that *C* and *D* become closed systems again, with their original Hamiltonians $H^{C}$ and $H^{D}$, which again acquire actual definite values, say, $\omega_{l}^{C}$ and $\omega_{l}^{D}$. In this way, “the event-time arises from the network of interaction relations between the systems that compose the whole closed universe”, and “the structure of that event-time is embodied in the internal structure of the Hamiltonian of that universe” ([61]: 22).

According to the PTI, the actual events are the transactions that actualize among the possible transactions between an emitter and several absorbers. According to the MHI, actual events are the acquisition of actual definite values by the Hamiltonians of closed systems at their constitution through interaction relations. Nevertheless, in the two cases the event-time is *the time of the realm of actuality*: it emerges from the network that the actual events constitute through their inter-relations. As a consequence, its nature is essentially *relational*. Moreover, since actualization is inherently indeterministic, it is not fixed by the theory; therefore, while the parameter-time is represented by the variable *t* of the Schrödinger equation, the event-time has *no theoretical representation*, it is a physical magnitude resulting from observable facts. Another point of agreement between the two interpretations is that the event-time, since it arises from the relationships between actual events, is not continuous like the parameter-time but rather *discrete*: there is no event-time between actual events.

**Figure 1:**
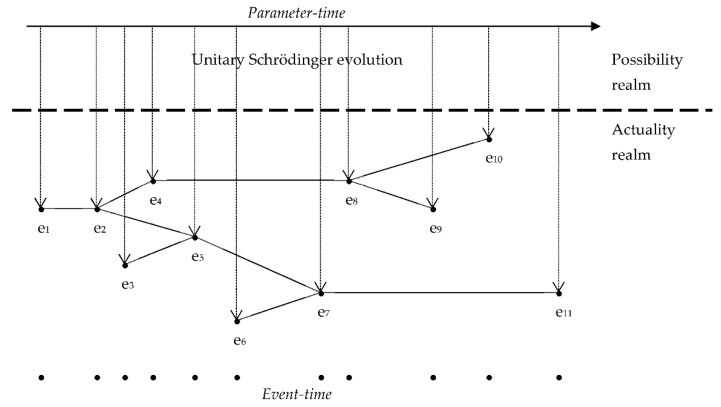
Relation between the possibility realm and the actuality realm.

The fact that the event-time has no theoretical representation in quantum mechanics is the counterpart of the fact that actualization is not accounted for by the theory. Quantum mechanics describes the evolution of the quantum state, which encodes possibilities; this means that quantum mechanics describes the domain of possibility. Then, just as the formalism does not determine which possibilities become actual, neither does it establish any theoretical link between the parameter-time and the event-time. The two times “come into contact” when certain possibilities spontaneously actualize and enter the domain of actuality (see Figure 1).

In summary, the distinction between the domain of possibility and the domain of actuality runs parallel to the distinction between parameter-time and event-time. Both ways of being and both temporalities are necessary to fully understand the quantum world. Moreover, the relational picture of time that emerges from both PTI and MHI appears as a promising approach to the so-called ‘problem of time in quantum gravity’ [52,53].

## 5. Final Remarks

Compared to the vast body of literature about quantum foundations, the philosophical inquiry about quantum possibility has been relatively sparse. In turn, the topic of time in quantum mechanics has attracted the interest of very few people in the large community of scholars working on the interpretation of quantum mechanics. In this paper we have argued that possibility and time are strongly linked in the quantum realm. On the one hand, we have advocated a possibilist interpretation of quantum possibility and probability, according to which both the domains of possibility and actuality complement each other in the constitution of reality, and neither of them is reducible to the other. In other words, quantum modality is not merely semantic or epistemic, but fundamentally ontological. On the other hand, we have argued that two essentially different notions of time must be distinguished in quantum mechanics: the parameter-time and the event-time. They correspond to the unitary Schrödinger evolution and to the occurrence of the observable events, respectively. Finally, we have argued for the close link between the two matters. The parameter-time is the time of the evolution of quantum possibilities, while the event-time arises from the relationships between actual events. Undoubtedly, this article does not exhaust the richness and complexity of this topic. However, we hope that it will become a starting point to promote interest in the deep relationships between modality and temporality.
